# Simvastatin inhibits cancer cell growth by inducing apoptosis correlated to activation of Bax and down-regulation of BCL-2 gene expression

**DOI:** 10.3892/ijo.2011.1273

**Published:** 2011-11-29

**Authors:** CARMINE SPAMPANATO, SALVATORE DE MARIA, MADDALENA SARNATARO, ELISABETTA GIORDANO, MARIO ZANFARDINO, SALVATORE BAIANO, MARIDELA CARTENÌ, FRANCO MORELLI

**Affiliations:** 1 Institute of Genetics and Biophysics A. Buzzati Traverso, CNR; 2 Department of Experimental Medicine, Medical School, II University of Naples, Naples, Italy

**Keywords:** simvastatin, cancer cell growth, apoptosis, Bax gene, BCL-2 gene

## Abstract

The statins (3-hydroxy-3-methylglutaryl coenzyme A reductase inhibitors) have been proven to be effective in lowering cholesterol and as anti-lipid agents against cardiovascular disease. Recent reports demonstrate an anticancer effect induced by the statins through inhibition of cell proliferation. Probably, these effects are due to suppression of the mevalonate pathway leading to the depletion of various downstream products that play an essential role in cell cycle progression, cell signaling and membrane integrity. To date, although many hypotheses have been proposed, the exact mechanism at the basis of cancer cell growth arrest induced by statins is not known. In this study, we have demonstrated that simvastatin, at a dose of 20 μM for 24–72 h, induced in cancer cells but not in normal cells precise features of apoptosis including increased DNA fragmentation while, at the molecular level simvastatin induced overexpression of the pro-apoptotic gene Bax together with an inhibition of BCL-2, the gene that has the well-known function of protecting cells from apoptosis. The simvastatin-mediated induction of apoptosis in similar cancer cells but not in normal cells is very interesting and may be at the basis of cancer therapy using statins, usually in combination with chemotherapy or to be used as a cancer protective drug. Simvastatin may, thus, play a dual prophylactic role as a lipid-lowering drug for the prevention of heart disease and as an anticancer agent to prevent certain types of cancers.

## Introduction

Statins, the 3-hydroxy-3-methylglutaryl coenzyme A (HMGCoA) reductase inhibitors, are a class of drugs that inhibit the rate-limiting step in the cholesterol biosynthetic pathway. Cholesterol is an important structural component of cell membranes and the physiological requirements derive by endogenous synthesis or exogenous supply. Increases in lipid levels lead to atherosclerosis and narrowing of the blood vessels, which in turn may affect the blood supply to the heart, brain and peripheral circulation, leading to morbidity or mortality (1).

Statins, by inhibiting cholesterol biosynthesis, emerged as principal agents in lowering the incidence of cardiovascular disease. However, it must be considered that any compound leading to depletion of cholesterol, which is the main structural component of cell membranes, affects various cellular events and impairs homeostasis. Statins, potent inhibitors of cholesterol synthesis, act by inhibiting 3-hydroxy-3-methylglutaryl CoA (HMG-CoA) reductase, which catalyzes the conversion of HMG-CoA to mevalonate (2). In addition to the cholesterol-lowering property, many biological effects of statins can be derived from cholesterol-independent pleiotropic mechanisms, which are likely a consequence of blocking intracellular signaling (3).

The role of statins extends beyond its lipid-lowering effects, as they are known to improve endothelial functions and participate in plaque stabilization, immune modulation and antioxidant activity and also act as anti-inflammatory and anticancer agents. These properties, together with a high safety profile, have made statins more attractive and continue to be a widely prescribed drug.

Their pleiotropic or cholesterol-independent effects at the cellular and molecular levels are highly related to numerous cellular functions, such as proliferation and differentiation. Treatment with simvastatin, mevastatin, atorvastatin or pravastatin induces morphological change and decrease cell proliferation. It has been observed that the use of simvastatin was more effective in cancer cells and embryonic stem cells (ESCs), in relation to normal cells. In ESC, the loss of self-renewal by simvastatin was characterized by marked down-regulation of several genes with function of ESC markers such as alkaline phosphatase, Oct4, Nanog, Rex-1 and SSEA-1. Simvastatin effects were selectively reversed by either mevalonate or its metabolite, geranylgeranyl pyrophosphate (GGPP), but not by cholesterol or farnesyl pyrophosphate (4).

Besides their use in the treatment of lipid disorders, statins have been studied for their anti-carcinogenic effects in several models, including carcinomas of the colon and rectum, prostate, breast, lung and skin (5,6). Many studies have shown the anti-proliferative and pro-apoptotic effects of statins to a greater degree in malignant than in non-malignant cells (7,8). Statins also can trigger different tumor cells to undergo apoptosis *in vitro* and suppress tumor growth (9,10).

In addition, substantial experimental and clinical evidence suggests that statins exhibit anticancer effects mediated by apoptosis and cell cycle arrest (11) through various signaling pathways. It has been hypothesized that statin-induced apoptosis is mediated by regulating BCL-2 family members involved in mitochondrial apoptosis pathway of various cells types (8,10,12,13). Moreover, statin attenuates the p53 stability response to DNA damage probably by phosphorylation of Mdm2. The tumor suppressor p53 is a key regulator of apoptosis, which has pro-apoptotic activity. Under stress conditions, p53 is stabilized and acts as a transcription factor that may increase the expression of pro-apoptotic target genes, such as Puma, Noxa, Bax and Bid (14). On the other hand, cytoplasmic p53 interacts with BCL-2 family member BCL-2 or BCL-XL, which results in activation and translocation of Bax and Bid to mitochondrial outer membrane. Moreover, p53 also translocates to the mitochondria to activate the mitochondrial apoptosis pathway (14–16). However, the molecular links between pro-apoptotic function of p53 and mitochondrial dysfunction in statin-induced apoptosis are not well understood.

Survivin is involved in apoptosis, and seems to be induced by simvastatin, the smallest member of the inhibitor of apoptosis protein (IAP) family. Survivin plays an important role not only in inhibiting apoptosis but also in regulating mitosis. Moreover, this gene is highly expressed in transformed cells and in most human cancers, including lung, breast, pancreatic and colon carcinomas, soft tissue sarcomas, brain tumors and hematologic malignancies (17).

Based on the above, we investigated the role of simvastatin in cancer cell growth inhibition showing the capacity of this drug to induce apoptosis and demonstrate that the induction of this process implicate the transcription up-regulation of Bax and down-regulation of BCL-2, two genes with important roles in the determination of programmed cell death.

## Materials and methods

### Cell culture

The MCF7 human breast cancer cells, SAEC human normal small airway epithelial cells, HepG2 human hepatocellular carcinoma cells, NCI-N87 human gastric cancer (NCI gastric cells) and NCiH12299 human non-small cell lung carcinoma (NCH lung) cells were purchased from American Type Culture Collection; the cells were grown at sub-confluent culture in Dulbecco’s modified Eagle medium or RPMI supplemented with L-glutamine, 100 U/ml penicillin, 10 μg/ml streptomycin and 10% fetal bovine serum, in 5% CO_2_ incubator at 37°C.

### Simvastatin treatment

Simvastatin (Calbiochem-Merck Co., Darmstadt, Germany) carboxylate forms represent a lipophilic 3-hidroxy-3-methylglutaryl coenzyme A (HMG-CoA) reductase inhibitor that blocks Ras function through the inhibition of farnesylation and inhibit glucose-induced Ca^2+^ channels in rat islet β cells and cell proliferation of human smooth muscle cells. This drug is soluble in dimethyl-sulfoxide (DMSO) and at a minor rate in ethanol.

In our experiments, simvastatin was dissolved in DMSO prepared in a 20-mM stock solution stored frozen at −20°C. For the experiment, the cells were plated and added with 20 μM simvastatin for 24–72 h in normal culture conditions. At the end of treatment, cells were washed with PBS and harvested by scraping in TRIzol reagent (Invitrogen, Carlsbad, CA, USA).

### RNA and cDNA synthesis

Total mRNA was extracted from simvastatin-treated cells according to Chomczynski and Sacchi method, using TRIzol reagent (Invitrogen) according to the manufacturer’s instructions and the integrity of purified RNA was verified using agarose gel electrophoresis.

For cDNA synthesis, total mRNA was extracted from simvastatin-treated cells according to Chomczynski and Sacchi; 2 μg of total RNA in a final volume of 25 μl was reverse-transcribed by Avian myeloblastosis virus (AMV) reverse transcriptase (Gibco-BRL) according to the manufacturer’s instruction, in the presence of random hexamer primers (Promega Corp., Madison, WI, USA) at 37°C per 60 min. The cDNA was controlled by PCR with housekeeping GAPDH or actin primers.

### DNA fragmentation analysis

The internucleosomal DNA fragmentation, a typical biochemical apoptotic event, was evaluated by agarose gel electrophoresis; 1×10^6^ cells were incubated in the standard culture medium at 37°C for different time in the presence of 20 μM simvastatin. At the end of the treatment, the cells were harvested by a cell scraper, centrifuged, washed in PBS and suspended in 100 μl TNE buffer (150 mM sodium chloride, 10 mM EDTA and 10 mM Tris-HCl pH 8). The cell suspensions were lysed with 3 volumes of lysis buffer (0.2% SDS and 50 μg/ml RNAse in TNE) and the lysate was incubated at 37°C for 1 h. High-molecular weight genomic DNA was extracted from lysates and analyzed using electrophoresis on 1.5% agarose gel in Tris-acetate-EDTA (TAE) (18). The induction of apoptosis by simvastatin was verified with the more sensible technique of TUNEL.

### TUNEL analysis

Cleavage of genomic DNA occurring during apoptosis may yield double-stranded, low-molecular weight DNA fragments as well single-strand nicks in high-molecular weight DNA. Those DNA strand breaks can be identified by labeling free 3′-OH termini using an enzymatic terminal deoxynucleotidyl transferase (TdT), which catalyzes tailing of fluorescein-labeled dUTP in the TUNEL (TdT-mediated dUTP nick end labeling) reaction. For TUNEL analysis, the Roche *in situ* cell death fluorescein detection kit (Roche Diagnostics, Mannheim, Germany) according to the manufacturer procedures was used. In brief, the plated cells were maintained in the presence of 20 μM simvastatin for 72 h, after the treatment cells were washed with PBS, fixed with 2% PFA, permeabilized with 0.1% Triton X-100 in 0.1% sodium citrate solution, added with TUNEL reaction mix and incubated in humidified chamber at 37°C for 60 min in the dark.

### PCR analysis

Three samples of the different cancer cell types were cultured for 48 h in DMEM 10% FCS containing 20 μM simvastatin. Control cell samples without simvastatin addition were maintained in identical culture conditions. Total RNA and cDNA synthesis was performed according to the procedure described. PCR analysis of both Bax and BCL-2 gene expression was performed using a GeneAmp PCR System 9700 (Applied Biosystems) and hot start Taq Gold (Applera). Actin was used as a housekeeping control gene. The sequences of primers utilized were as follows: Bax forward, 5′-CCA GCT CTG AGC AGA TCA TG-3′ and reverse, 5′-TGC TGG CAA AGT AGA AAA GG-3′; BCL-2 forward, 5′-GAC TTC GCC GAG ATG TCC AG-3′ and reverse, 5′-CAG GTG CCG GTT CAG GTA CT-3′; Actin forward, 5′-GAC TAC CTC ATG AAG ATC CT-3′ and reverse, 5′-GCT TGC TGA TCC ACA TCT GC-3′. PCR conditions were as follows: initial denaturation at 95°C for 10 min followed by 35 cycles: 95°C for 45 sec, 54°C for 45 sec and 72°C for 45 sec with a final extension at 72°C for 10 min. The amplification products were analyzed on a 1% agarose gel in 0.5X Tris Borate EDTA (TBE) buffer to control the amplicon lengths.

### Real-time PCR

Real-time PCR analysis of BCL-2 and Bax gene expression was performed using the iCycler^®^ apparatus (Bio-Rad, Hercules, CA) with sequence-specific primer pairs for the genes tested. The housekeeping gene actin was used for the normalization.

The primers used were the following: β-actin forward, 5′-GAC TAC CTC ATG AAG ATC CT-3′ and reverse, 5′-GCT TGC TGA TCC ACA TCT GC-3′; hBax forward, 5′-CCA GCT CTG AGC AGA TCA TG-3′ and reverse, 5′-TGC TGG CAA AGT AGA AAA GG-3′; and hBCL-2 forward, 5′-GAC TTC GCC GAG ATG TCC AG-3′ and reverse, 5′-CAG GTG CCG GTT CAG GTA CT-3′. The cDNA was serially diluted and every dilution was run at least in triplicate. The real-time PCR analysis was performed as follows: initial denaturation step, 95°C for 3 min followed by 50 cycles of denaturation at 95°C for 1 sec; annealing, 10 sec at 50°C; and elongation, 8 sec at 72°C. The IQ SYBR-Green SuperMix (Bio-Rad) was used for real-time PCR monitoring of the amplification. Briefly, amplification was performed in a total volume of 30 μl; the reaction mix was performed with 15 μl of 2X IQ SYBR-Green SuperMix, 0.5 μl of each primer (16 μM) and 2 μl of cDNA (or water as control, was always included). The real-time PCR products were run on 2% agarose gel in TAE (standard Tris-acetate-EDTA electrophoretic buffer). The amplicons of expected size were extracted, purified and controlled for sequences by Biogem DNA Sequencing Core (Biogem, Naples, Italy). The sequence analysis do not show any differences from Bax and BCL-2 gene sequences already deposited in GenBank. Results were evaluated by iCycler iQ Real-Time Detection System Software^®^ (Bio-Rad). Data were calculated on the basis of the threshold cycle (Ct) value. The expression of the analyzed genes was first normalized with respect to β-actin. The relative amount of mRNA on the y-axis in the graph was expressed as the inverse of Ct normalized values and multiplied by 100 (1/Ct × 100). The value obtained was proportional to mRNA expression.

### Western blot analysis of Bax and BCL-2 in simvastatin-treated MCF7 cells

MCF7 breast cancer cells and SAEC human normal small airway epithelial cells were collected in 1.5-ml tubes, washed with PBS and lysed in 0.4 ml of lysis buffer (0.06 M Tris-HCl, pH 6.8, 10% glycerol, 2% SDS, 5% β-mercaptoethanol and 0.0025% bromophenol blue). DNA was sheared by a needle and the solution was heated at 95°C for 5 min and centrifuged at 15000 g for 2 min. The total protein was quantified and loaded on SDS-polyacrylamide gel, run at 40 mA and transferred to nitrocellulose by electroblotting. Filters were blocked with 5% non-fat milk in TBS/Tween-20 buffer (137 mM NaCl, 20 mM Tris-HCl, pH 7.4, 0.1% Tween-20) before incubation with antibodies against Bax (mouse monoclonal dilution 1:200; Santa Cruz Biotechnology, Santa Cruz, CA, USA), BCL-2 (mouse monoclonal dilution 1:200; Santa Cruz Biotechnology) or β-actin (mouse monoclonal dilution 1:30000; Sigma-Aldrich, Munich, Germany), followed by horseradish peroxidase-conjugated anti-mouse-IgG secondary antibody (dilution 1:30000; Promega Corp.) dissolved in 1% non-fat milk in TBS/Tween-20. Immune complex were detected by the Super Signal West Pico Chemiluminescent Kit (Pierce, Rockford, IL, USA) and exposed to X-ray film (Kodak, Wiesbaden, Germany).

### Statistics

Each experiment was repeated three times. Results are expressed as mean ± SEM and the effects were compared with untreated control cells on the same plate. Paired t-test were used to analyze the effect of simvastatin; P<0.05 was considered significant.

## Results

### DNA laddering

**Figure 1:**
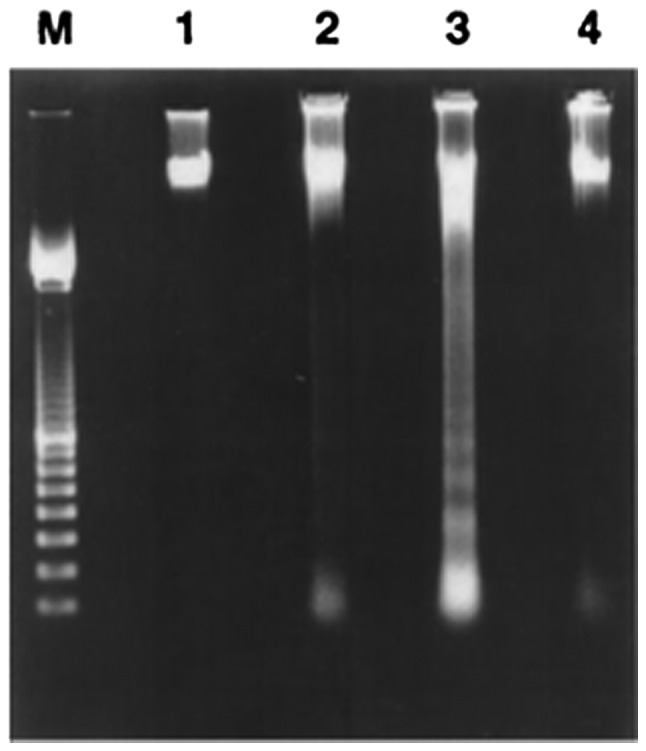
Internucleosomal DNA fragmentation. Treatment of MCF7 cells with 20 μM simvastatin for 24 h induces internucleosomal DNA fragmentation. DNA was extracted after treatment with simvastatin for 1 day (lane 2) or 3 days (lane 3). Lane 1, untreated MCF7 cells; lane 4, SAEC human normal small airway epithelial cells treated with simvastatin 20 μM for 3 days. Lane M, 100-bp ladder (Boehringer, Mannheim, Germany) used as a size marker.

**Figure 2:**
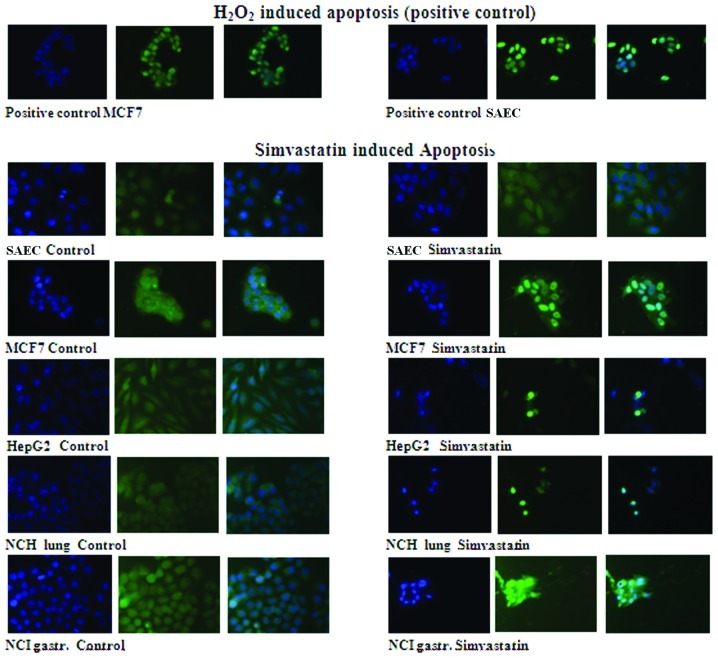
TUNEL apoptosis analysis. Positive control of TUNEL reaction is obtained by inducing apoptosis with hydrogen peroxide in breast cancer MCF7 cells and in SAEC human normal small airway epithelial cells where a clear fluorescein green coloration appears inside the nuclei. No sign of apoptosis is induced by simvastatin in non-transformed human fibroblast. In breast cancer MCF7, hepatocellular HepG2, lung carcinoma NCH lung, and human gastric cancer cells NCI gastric showed strong apoptosis, as indicated by the clear green fluorescence in the cell nuclei.

Simvastatin treatment induces DNA fragmentation. Internucleosomal DNA fragmentation is a classic biochemical event occurring in cells undergoing apoptosis. DNA degradation, characterized by a typical electrophoretic ladder, was found in breast cancer MCF7 cells treated for 1 or 3 days with simvastatin 20 μM, whereas it did not occur in cells without treatment (Fig. 1) or in SAEC human normal small airway epithelial cells treated with the same simvastatin concentration for 3 days. The induction of the apoptotic event was time-dependent and is minimal after 1 day of treatment but reached maximum intensity in cancer cells treated with 20 μM simvastatin for 3 days (Fig. 1). *In situ* DNA fragmentation also was demonstrated by the TUNEL assay, which showed the occurrence of DNA fragmentation (presence of green nuclei) in several cancer cell types but not in fibroblast cells treated for 2 days with 20 μM of simvastatin (Fig. 2).

### TUNEL apoptosis analysis

The TUNEL analysis clearly showed that 72-h treatment with 20 μM simvastatin induces a well-defined apoptosis in five different types of human cancer cells but not in non-tumoral human SAEC that do not show apoptotic signs either when the drug treatment was prolonged >72 h. As positive control of TUNEL reaction, SAEC normal epithelial and breast cancer MCF7 cells, in which the apoptosis was induced by 30-min treatment with hydrogen peroxide, was used.

For all cell lines treated with simvastatin, the nuclei DAPI coloration (blue) and the fluorescein (green) coloration of apoptotic nuclei were visible, and the composite image was observed (Fig. 2).

The composite coloration clearly appears as the fluorescein is localized only in the nuclei, showing that 72-h treatment with 20 μM simvastatin induces strong apoptosis in cancer cells but not in normal cells. SAEC control cells after 3 days of treatment did not show apoptotic coloration in the nuclei. On the contrary, the apoptosis induction with a strong green coloration localized in the inner nuclei is clearly evident in MCF7 breast cancer cells. Similar results appear in hepatocellular carcinoma HepG2 cells, lung carcinoma NCH lung cells and gastric cancer NCI gastric cells; in these cancer cell lines, there was a high mortality after 72-h drug treatment and only a minor number of cells remain attached on the cell culture plate. In these residual cells, a very strong nuclei green coloration due to the well-evident apoptosis induction was observed (Fig. 2).

### PCR analysis

**Figure 3:**
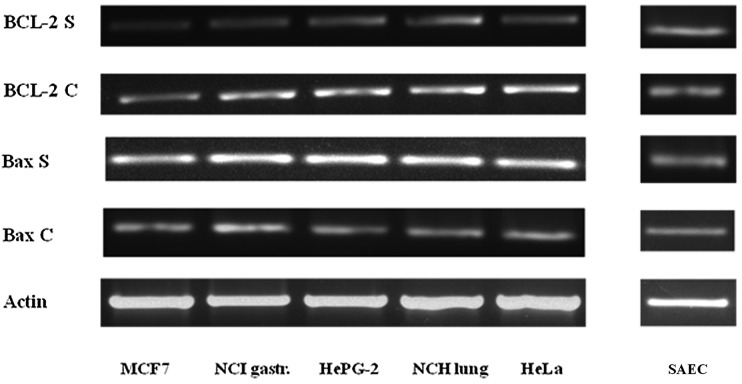
PCR analysis of Bax and BCL-2 expression. The PCR expression profile of Bax and BCL-2 genes demonstrate that, in all cancer cells, there is an increase in Bax expression and a diminution in the expression of BCL-2 respect to the untreated control cells. On the contrary, there are no differences in the expression of these genes in the SAEC human normal small airway epithelial cells. Actin is reported as the internal control.

The Bax and BCL-2 PCR analysis demonstrate that the simvastatin treatment, in five different types of cancer cells, induce apoptotic death, characterized by an increase in the expression of the pro-apoptotic gene Bax and, at the same time, a decrease in the expression of the anti-apoptotic gene BCL-2 compared to the untreated control cells. In the SAEC non-cancerous cells, there was no difference in the expression of these two genes after the same simvastatin treatment as shown in Fig. 3.

### Real-time PCR analysis

**Figure 4:**
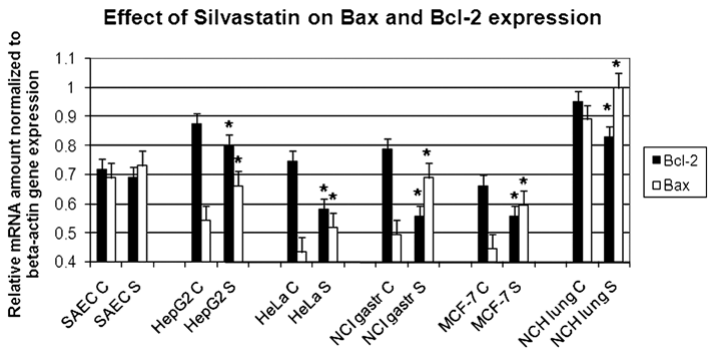
Real-time PCR analysis of Bax and BCL-2 genes. The real-time PCR analysis of Bax and BCL-2 genes demonstrate that, in all simvastatin-treated cancer cells (S), there is an increase in Bax expression and a diminution in the expression of BCL-2 with respect to controlling untreated cells (C). On the contrary, there are no differences in the expression of these genes in the SAEC normal small airway epithelial cells. The values have been normalized with respect to actin. The graph represents three independent experiments with each bar as mean ± SD. ^*^P<0.05 compared to control group.

The variation of apoptotic gene expression, already demonstrated in the semi-quantitative PCR analysis, is clearly confirmed by real-time amplification as shown in Fig. 4.

In all the cancer cell lines analyzed, the simvastatin treatment appears to reduce anti-apoptotic BCL-2 expression and increase the transcription of Bax pro-apoptotic gene. The values have been normalized with respect to actin. The graph represents three independent experiments with each bar as mean ± SD. P<0.05 compared to control group.

### Western blot analysis of Bax and BCL-2 in simvastatin-treated MCF7 cells

**Figure 5:**
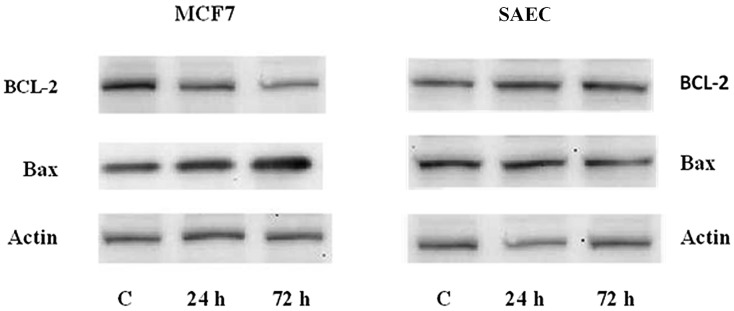
Apoptosis protein analysis. BCL-2 and Bax protein was determined by Western blot analysis after 24 and 72 h of treatment with 20 μM simvastatin on MCF7 breast cancer cells and SAEC human normal small airway epithelial cells. Actin was reported as internal control. C, the cells are maintained for 72 h without drug addition. It is evident that the incubation with simvastatin caused time-dependent increase in Bax expression and decrease in BCL-2 protein expression only in MCF7 cancer cells.

The induction of apoptosis during the simvastatin treatment was evinced in the variation of the expression of proteins responsible for the regulation of apoptosis mechanism. We have analyzed Bax and BCL-2, the two principal genes involved in the apoptosis control, and have demonstrated that at the basis of apoptosis induction, there is an up-regulation of Bax protein, which has a well-known pro-apoptotic effect, and a down-regulation of BCL-2, which is known to protect the cells from apoptosis. In this experiment, we have demonstrated that in MCF7 breast cancer cells, the quantity of apoptotic proteins vary, increasing the time of simvastatin presence in the cell culture. On the contrary, in non-malignant SAEC cells, there are no variations in Bax and BCL-2 protein quantity (Fig. 5).

## Discussion

Many studies show statins to be beneficial as anticancer agents (19). Their anti-tumor effects may be due to the involvement of important biological processes, such as inhibition of cell proliferation, promotion of apoptosis, inhibition of angiogenesis, prevention of metastasis, improvement of immunity or possibly targeting the cancer stem cell population (20).

In the present study, we investigated the anti-proliferative effects of simvastatin on several types of cancer cells. According to our results, the anticancer cell growth inhibition is due to the deregulation of apoptosis induction. Apoptosis plays a key role in the pathogenesis of cancers and the genes relating to this process are focus of interest in the studies of cancer onset and progression. It is well-known that Bax and BCL-2 are transcriptional targets for the tumor suppression protein p53 (17), which is responsible for the induction of cell cycle arrest and/or apoptosis in response to DNA damage (21).

The progression of cancer mainly depends on the balance between the pro-apoptotic protein such as Bax and anti-apoptotic protein such as BCL-2 (22). Moreover, p53-independent mitochondrial-mediated apoptosis has been reported following lovastatin exposure in a mouse mammary carcinoma (23).

It has been proposed that simvastatin regulates BCL-2 protein levels through the induction of ET-1, which involves the transcription factor NFATc3 binding to the BCL-2 promoter. This hypothesis is based on the evidence that exogenous ET-1 significantly increased BCL-2 protein levels in neuroblastoma cells and this effect was attenuated in the presence of ETA/B receptor antagonists (24).

In our study, we have demonstrated that the incubation of several lines of human cancer cells, of different histology, with 20 μM simvastatin caused a significant increase in Bax expression and a decrease in BCL-2, both at mRNA and protein levels, and as a consequence of this gene expression deregulation, we observed strong apoptosis induction, after addition of simvastatin, in all the cancer cell lines examined. As determined with the TUNEL analysis, the clearest results were observed in MCF7 breast cancer cells and in NCI gastric cancer cells with clear fluorescence inside the cell nuclei. In lung carcinoma and in hepatic carcinoma cells, apoptotic death was very strong and the small number of cells that remain attached to the plate showing a very strong fluorescein coloration in the nuclei. On the contrary, the non-cancerous fibroblasts are not sensitive to simvastatin and do not show any sign of apoptosis, even after 3 days of exposure to the drug. This different sensitivity between the normal and cancer cells may have important implications on the possibility to use this drug, already widely used in the therapy of hypercholesterolemia, as protection against cancer progression or cancer prevention.

In conclusion, these studies indicate that statins are able to induce apoptosis in different cancer cell lines and the mechanism at the basis of induction of this important process involves the regulation of Bax and BCL-2 gene expression. The confirmation of these effects with experiments on animals *in vivo*, to verify if, at therapeutic or at higher doses, simvastatin may induce a regression in tumor mass, preventively induced in mice with syngenic cancer cells or with carcinogenic chemicals, is important. These experiments are ongoing in our laboratory.

There is an increasing interest in cancer protection and in all the drugs that at low dose, alone or in combination with different modes of action and low toxicity function as chemopreventive agents. Therefore, we are also studying other molecules that are generally used for the treatment of well-known pathology and which present interesting effects on cancer cell proliferation.
